# Prognostic Value of Serum Galectin-3 for Survival in Patients with Cardiac Light-Chain Amyloidosis

**DOI:** 10.3390/jcdd11070202

**Published:** 2024-06-29

**Authors:** Xinglin Yang, Jin Huang, Jinghong Zhang, Jian Li, Zhuang Tian

**Affiliations:** 1 Department of Cardiology, Peking Union Medical College Hospital, Chinese Academy of Medical Sciences & Peking Union Medical College, Beijing 100730, China; yangxinglindz@163.com; 2 Department of Internal Medicine, Peking Union Medical College Hospital, Chinese Academy of Medical Sciences & Peking Union Medical College, Beijing 100730, China; huangjin@pumch.cn; 3 Central Clinical School, Monash University, Melbourne, VIC 3004, Australia; jinghongz.anderson@gmail.com; 4 Department of Hematology, Peking Union Medical College Hospital, Chinese Academy of Medical Sciences & Peking Union Medical College, Beijing 100730, China; lijian@pumch.cn

**Keywords:** galectin-3, cardiac light-chain amyloidosis, overall survival, heart failure

## Abstract

Background: Amyloid light-chain (AL) amyloidosis is a multisystem disorder, with cardiac amyloid infiltration being a prevalent manifestation. This study aimed to explore the prognostic value of galectin-3 (Gal-3), a soluble marker associated with fibrosis, inflammation, heart failure, and kidney injury, in patients with cardiac AL amyloidosis. Methods: A total of 60 patients who were diagnosed with cardiac AL amyloidosis from January 2015 to May 2018 were enrolled. The prognostic value of Gal-3 was assessed. Receiver operating characteristic (ROC) curves were used to evaluate the predictive accuracy of Gal-3. A Gal-3 cut-off value was identified to predict survival rates. Results: The ROC curves demonstrated a moderate predictive accuracy of Gal-3 for 0.5- and 5-year survival, with area under the curve (AUC) values of 0.722 and 0.788, respectively. A Gal-3 cut-off value of 15.154 ng/mL was found to predict survival. Kaplan–Meier survival analysis revealed a significant difference in mean overall survival between patients with Gal-3 levels below and above the established cut-off (69.2 months versus 42.1 months, respectively; *p* = 0.036). Multivariate analysis confirmed that Gal-3 > 15.154 ng/mL remained an independent predictor of survival (HR 2.451, 95% CI 1.017–5.910, *p* = 0.046). Conclusions: This study suggests that Gal-3 holds independent prognostic value for survival in patients with cardiac AL amyloidosis. Gal-3 could potentially enhance the prognostic capabilities of the current soluble markers, thereby improving the management of cardiac AL amyloidosis. However, further validation in larger prospective studies is warranted.

## 1. Introduction

Amyloid light-chain (AL) amyloidosis is a multisystem disorder that frequently impacts various organs and systems, including the liver, kidney, spleen, lung, and heart, as well as the autonomic and peripheral nervous systems. Cardiac amyloid infiltration is a prevalent manifestation, occurring in up to approximately 75 percent of patients diagnosed with AL amyloidosis [1]. This condition arises when misfolded immunoglobulin light chains aggregate to form amyloid fibrils which subsequently deposit in the heart. The degree of cardiac dysfunction serves as the principal determinant of morbidity and mortality in patients with AL amyloidosis.

Several biomarkers have been identified as prognostic factors in AL amyloidosis, including cardiac troponins (cTnT or cTnI), N-terminal pro-B-type natriuretic peptide (NT-proBNP), and free light-chain (FLC) levels. These biomarkers have been incorporated into staging systems, such as the Mayo Clinic staging system, which stratifies patients based on the levels of NT-proBNP, cTnT, and the difference between the involved and uninvolved FLC (dFLC). The Mayo stage III has been further subdivided into stages IIIA and IIIB based on the presence or absence of significant right ventricular dysfunction and an NT-proBNP cut-off at 8500 ng/L. Patients with Mayo stage IIIB have been shown to have the worst prognosis [2,3]. The elevation of different markers is driven by distinct underlying mechanisms, rendering them complementary to each other. While these biomarkers have demonstrated significant prognostic value, the quest for additional markers remains essential to further refine risk stratification and pave the way for personalized treatment strategies tailored to individual patient needs. Galectin-3 (Gal-3), which is a beta-galactoside-binding lectin implicated in fibrosis and inflammation, plays a critical role in the progression of heart failure and kidney injury. Gal-3 elevation has been found to be associated with an increased mortality risk in AL amyloidosis patients with renal involvement [4]. However, the correlation between Gal-3 levels and prognosis in patients with cardiac AL amyloidosis remains unknown.

In this study, the role of Gal-3 in patients with cardiac AL amyloidosis was investigated. The objective was to determine whether Gal-3 holds any additional prognostic value in addition to the other recognized risk factors.

## 2. Materials and Methods

### 2.1. Study Population

Patients admitted to our hospital with a new diagnosis of primary AL amyloidosis from January 2015 to May 2018 were enrolled in this study. The diagnosis was established by the presence of Congo red-positive fibrillar deposits in biopsy samples, and the AL subtype was identified using a variety of methods, including immunohistochemistry, immunofluorescence, and proteomics. The diagnostic biopsy sites were as follows: the myocardium in 20 patients (33.33%), kidney in 17 patients (28.33%), subcutaneous fat in 9 patients (15%), tongue in 6 patients (10%), bone marrow in 5 patients (8.33%), rectum in 2 patients (3.33%), lung in 1 patient (1.67%), and nerve in 1 patient (1.67%). Patients who did not have cardiac involvement in disease manifestation or had evidence of active infection were excluded from this study. Cardiac involvement was defined as a mean left ventricular wall thickness exceeding 12 mm on echocardiography and no other cause that could explain NT-proBNP levels exceeding 332 pg/mL in the absence of renal failure or atrial fibrillation [5]. In patients with concurrent hypertension, we required the presence of additional characteristic features of cardiac amyloidosis, including low voltage on ECG, right ventricular or heart valvular thickening, longitudinal strain alteration, and a typical late gadolinium enhancement pattern or increased extracellular volume on cardiac magnetic resonance imaging, to ensure diagnostic accuracy. Ultimately, 60 eligible patients were included. Following the diagnosis of AL amyloidosis, all patients received initial treatment with cyclophosphamide, bortezomib, and dexamethasone (CyBorD regimen). Patients were followed up through regular outpatient visits or telephone consultations, which were performed by the physicians in our research team till July 2023. These physicians, who are experienced in managing patients with AL amyloidosis, assessed the patients’ clinical status, monitored their response to treatment, and documented any adverse events or outcomes. This study was approved by the Ethics Committee of our hospital and was conducted in accordance with the principles of the Declaration of Helsinki. Informed consent was secured from the patients for their inclusion in this study.

### 2.2. Laboratory Methods

Blood samples for Gal-3 measurement were collected from patients at the time of diagnosis, prior to the initiation of any specific therapy. The samples were obtained using a 5 mL vacutainer and centrifuged at a speed of 3000 revolutions per minute for a duration of 5 min. The extracted serum was stored at a temperature of −80 °C. All samples underwent analysis using the same batch of reagent. Gal-3 serum levels were quantified utilizing a commercial Enzyme-Linked Immunosorbent Assay on an automated plate reader.

### 2.3. Statistical Analysis

Baseline characteristics, including demographic data and initial measurements, are presented as the mean ± standard deviation (SD) for normally distributed continuous variables, and as the median with interquartile range (IQR) for continuous variables exhibiting skewed distribution. Student’s *t*-test or the Mann–Whitney U-test was utilized to analyze continuous variables between two groups, depending on the data distribution. Categorical variables are summarized as counts and percentages and were compared using the chi-squared (χ^2^) test. Overall survival (OS) was defined as the time from diagnosis to death, and patients alive at the time of the last follow-up were censored on that date. Survival curves were constructed using the Kaplan–Meier method, and these curves were compared using the log-rank test. The optimal cut-off points were identified using logistic regression. Multivariate analysis was conducted using logistic regression and Cox proportional hazards. Statistical significance was set at a *p* < 0.05. All data were subjected to statistical analysis using GraphPad Prism (version 9.0; GraphPad Software, San Diego, CA, USA), SPSS (version 26.0; IBM, Armonk, NY, USA), and R software (version 4.2.2; R Foundation for Statistical Computing, Vienna, Austria), with the analysis performed by two investigators.

## 3. Results

The demographic and baseline characteristics of the 60 patients involved in this study are summarized in Table 1. The median age of the study population was 60.02 years, with males comprising 60% of the sample. The mean serum Gal-3 level was observed to be 14.28 ± 5.29 ng/mL.

**Figure 1:**
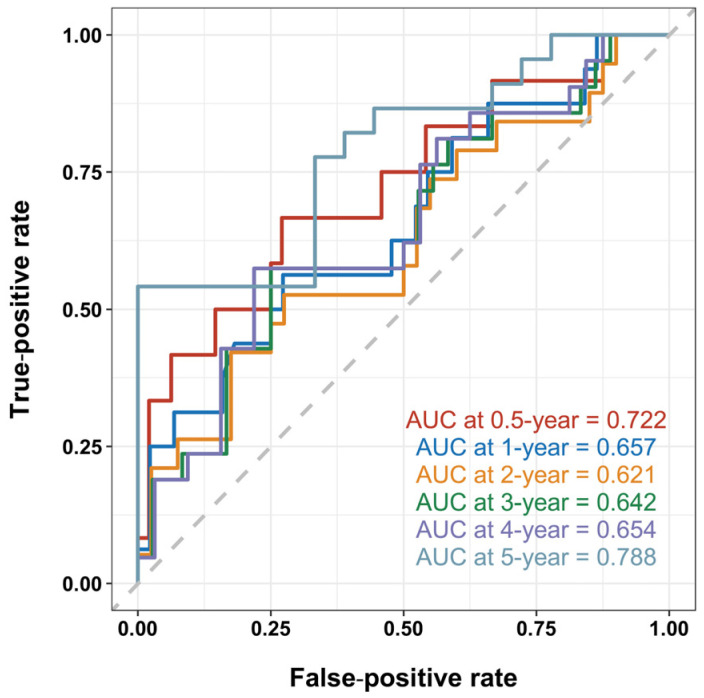
Time-dependent receiver operating characteristic (ROC) curve for galectin-3 (Gal-3). The dashed diagonal line from the bottom-left to the top-right corner represents the performance of a random classifier, serving as a reference baseline. The area under the curve (AUC) values for 0.5- and 5-year survival were 0.722 and 0.788, respectively. The AUC values for 1-, 2-, 3-, and 4-year survival were all less than 0.7.

To evaluate the prognostic value of serum Gal-3 in mortality for cardiac AL amyloidosis, a time-dependent receiver operating characteristic (ROC) curve was employed. The area under the curve (AUC) values for 0.5- and 5-year survival rates were 0.722 and 0.788, respectively, suggesting moderate predictive accuracy. However, the AUC values for 1-, 2-, 3-, and 4-year survival rates were all smaller than 0.7, indicating a lower accuracy at these time points (Figure 1). The ROC curve analysis identified predictive cut-off values of Gal-3 for 0.5- and 5-year OS to be 15.154 ng/mL and 10.088 ng/mL, respectively. The 0.5-year cut-off of 15.154 ng/mL was chosen over the 5-year cut-off, as early risk stratification is more clinically relevant for guiding immediate treatment decisions and monitoring in these patients with rapidly progressing cardiac AL amyloidosis.

**Figure 2:**
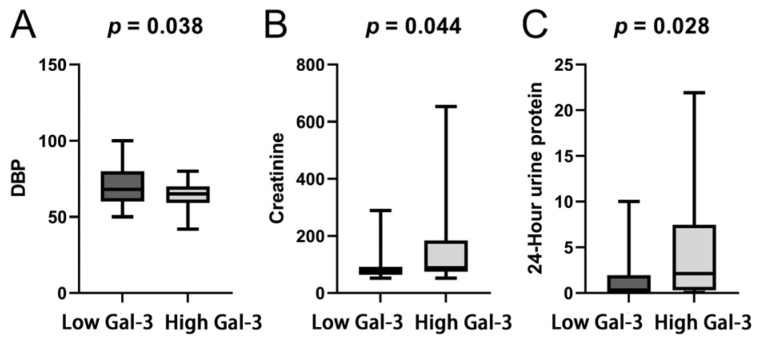
Baseline characteristics of significant differences between patient groups with low levels (≤15.154 ng/mL) and high levels (>15.154 ng/mL) of galectin-3 (Gal-3). (**A**), diastolic blood pressure (DBP; *p* = 0.038). (**B**), creatinine (*p* = 0.044). (**C**), 24-h urine protein (*p* = 0.028).

We selected 15.154 ng/mL as the threshold to divide patients into two groups based on their serum Gal-3 levels being either higher or lower than 15.154 ng/mL. Significant differences were observed in baseline characteristics between the patient groups with low levels (≤15.154 ng/mL) and high levels (>15.154 ng/mL) of Gal-3 (Table 1). These characteristics included diastolic blood pressure (DBP; *p* = 0.038), creatinine (*p* = 0.044), and 24-h urine protein (*p* = 0.028; Figure 2A, Figure 2B, and Figure 2C, respectively). Patients with higher Gal-3 levels were found to be more likely to have lower DBP, higher creatinine, and increased 24-h urine protein levels.

**Figure 3:**
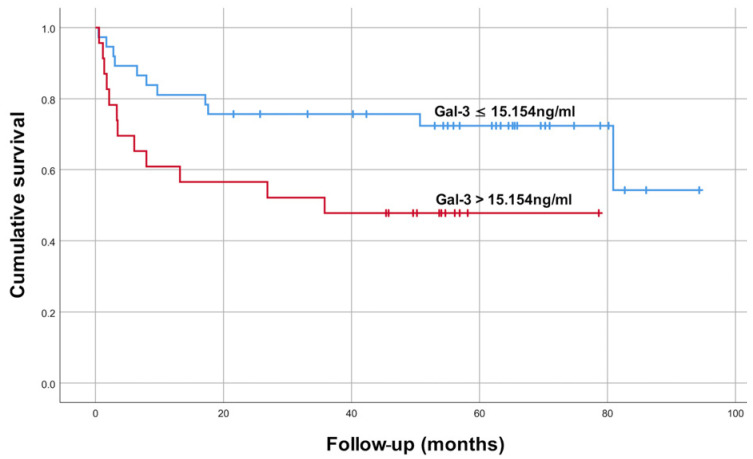
Survival based on whether galectin-3 (Gal-3) was below (red) or above (blue) the cut-off point of 15.154 ng/mL. The respective median overall survivals were 69.2 months (95% confidence interval [CI] 57.0, 81.7) and 42.1 months (95% CI 27.5, 56.8), *p* = 0.036.

Survival analysis was conducted using the Kaplan–Meier method to investigate the impact of Gal-3 levels on the survival rates of patients with cardiac AL amyloidosis (Figure 3). The respective mean OS for the two groups was 69.2 months (95% confidence interval [CI] 57.0, 81.7) and 42.1 months (95% CI 27.5, 56.8), with a significant difference between the groups (*p* = 0.036).

The multivariate Cox regression model incorporated the primary variable of interest, Gal-3 level (>15.154 ng/mL), along with parameters that differed significantly between groups in the baseline analysis (decreased DBP, increased serum creatinine, and increased 24-h urine protein levels). Additional variables considered potentially important prognostic factors included age (>60 years), gender (male), advanced NYHA functional class (III/IV), advanced Mayo 2012 stage (III/IV), and no treatment response. Upon multivariate analysis, high Gal-3 level (HR 2.734, 95% CI 1.036–7.216, *p* = 0.042), advanced Mayo 2012 stage (HR 4.184, 95% CI 1.308–13.390, *p* = 0.016), and no treatment response (HR 4.434, 95% CI 1.573–12.500, *p* = 0.005) demonstrated an independent association with survival outcomes. The other factors analyzed were not independently prognostic when adjusted for in the model (Table 2).

## 4. Discussion

This study evaluated the prognostic value of serum Gal-3 in 60 patients with cardiac AL amyloidosis. The ROC curve indicated a moderate predictive accuracy of Gal-3 for 0.5- and 5-year survival. A Gal-3 cut-off value of 15.154 ng/mL was identified. Patients with Gal-3 levels above the cut-off exhibited significant differences in baseline characteristics, including lower DBP, higher creatinine, and increased 24-h urine protein levels. Kaplan–Meier survival analysis indicated shorter mean OS for patients with higher Gal-3 levels. Importantly, the prognostic value of Gal-3 was found to be independent of age, gender, NYHA functional class, Mayo 2012 cardiac biomarker staging system, and the other three variables that showed significant inter-group differences.

AL amyloidosis, also known as primary amyloidosis, is the predominant type of systemic amyloidosis. The underlying etiology involves clonal plasma cell expansion, which produces amyloidogenic immunoglobulin light chains. These light chains aggregate to form insoluble fibrils that deposit in tissues, leading to organ dysfunction. The heart is one of the most commonly affected organs in AL amyloidosis [6]. Cardiac dysfunction in AL amyloidosis is primarily attributed to two factors, which are extracellular infiltration of the myocardium and the direct toxic effects of circulating light chains [7]. Recent studies have identified additional contributing mechanisms such as fibrosis and inflammatory pathways [8,9]. It is worth noting that the median survival in AL amyloidosis is contingent on the cardiac stage of the disease at diagnosis [3]. Hence, it is crucial to develop a prognostic evaluation system based on target organ damage and plasma cell dyscrasia in order to optimize treatment and enhance OS in patients with cardiac AL amyloidosis. In this context, the Mayo 2004 and 2012 staging systems, which were designed to estimate cardiac involvement and long-term survival in AL amyloidosis, are widely utilized. Furthermore, emerging evidence indicates that introducing new variables could enhance the predictive value of the staging system [10].

Galectins, a family of widely expressed β-galactoside-binding lectins, can modulate fundamental cellular functions, such as cell-to-cell and cell-to-matrix interactions, cell growth and differentiation, tissue regeneration, and regulation of immune cell activities [11]. Galectins are classified based on the number and function of their carbohydrate recognition domains. Gal-3, a member of this family and an approximately 30 kDa chimera-type galectin, is expressed by various immune cells, including mast cells, histiocytes, and macrophages. These cells are associated with the mononuclear phagocytic system in diverse tissues. Gal-3 has been shown to play a crucial role in various physiological functions, such as cell growth and differentiation, macrophage activation, angiogenesis, apoptosis, and antimicrobial activity, and also acts as a mediator of local inflammatory responses in many pathological conditions [12,13]. Given that Gal-3 is readily expressed on the cell surface and easily secreted into biological fluids from injured and inflammatory cells, recent studies suggest that serum Gal-3 could serve as a marker for cardiac disorders, such as cardiac inflammation and fibrosis [14].

In a study conducted by Li et al., it was found that baseline serum Gal-3 levels exceeding 20.24 ng/mL could function as an independent predictor of all-cause mortality in patients with renal AL amyloidosis [4]. In contrast, another recent study that included 502 patients diagnosed with AL amyloidosis (with 69% cardiac involvement and 55% renal involvement) failed to substantiate the predictive value of Gal-3 in a multivariate analysis [15]. Our study, with a focus on patients with cardiac AL amyloidosis, yielded an outcome that was consistent with the findings of the renal-focused study. The divergence in these findings might be attributed to the heterogeneity of the study populations. Previous reports have suggested that the Gal-3 pathway is predominantly associated with renal and cardiac dysfunction or damage [16,17]. Gal-3 appears to be primarily correlated with prognosis in instances where the heart or kidneys are affected. Our study found that patients with higher Gal-3 levels were more likely to have lower DBP, possibly due to greater cardiac dysfunction and autonomic dysfunction. The higher creatinine levels and proteinuria in the Gal-3 high group suggest a greater extent of renal damage. However, estimated glomerular filtration rate (eGFR) did not show significant differences between the groups, possibly due to eGFR being a composite measure influenced by various factors that our sample size may not adequately capture. Interestingly, we did not find significant differences in dFLC levels between the high- and low-Gal-3 groups. While dFLC is an important biomarker for the diagnosis and assessment of AL amyloidosis, its variations may not always synchronize with changes in Gal-3 expression. Despite these, our findings provide valuable insights into the role of Gal-3 in renal and cardiac complications in AL amyloidosis, potentially leading to an improved management strategy for this condition.

The application of ROC curve analysis revealed that Gal-3 could serve as a moderate predictor for both 0.5- and 5-year survival, as demonstrated by the AUC values. This finding may be attributed to the inherent characteristics of AL amyloidosis, a disease that presents a dichotomy in terms of short- and long-term survival. Patients with severe cardiac involvement often deteriorate within a short period of time, while others can survive for more than 5 years as a result of appropriate treatment. These findings are consistent with a previous study in which the median survival was found to be at 5 years despite up to 43% of patients not surviving past the first year [18]. For those with advanced cardiac disease, the median survival dwindles to less than 6 months [19]. Thus, it appears that Gal-3 could be a valuable tool in predicting both short- and long-term survival outcomes.

Further supporting this notion, Kaplan–Meier survival analysis demonstrated a significant correlation between higher Gal-3 levels and reduced survival times. This finding consolidates the prognostic value of Gal-3 in patients with cardiac AL amyloidosis. Remarkably, the multivariate model indicated that serum Gal-3 levels have the potential to serve as an independent mortality predictor in patients with cardiac AL amyloidosis.

It should be acknowledged that Gal-3 is not a specific marker for AL amyloidosis but rather an indicator of organ dysfunction in general. Elevated Gal-3 levels have been observed in various cardiovascular diseases, such as heart failure and atrial fibrillation, as well as in renal dysfunction. In the context of cardiac AL amyloidosis, the prognostic value of Gal-3 may be attributed to its ability to reflect the overall severity of cardiac and renal involvement. Nonetheless, our findings suggest that Gal-3 could provide additional prognostic information beyond the currently used markers in cardiac AL amyloidosis.

This study has several limitations that warrant consideration. Firstly, it is important to note that all patients in our study received uniform initial treatment with the CyBorD regimen following their diagnosis. However, at the time of this study, newer treatment options such as daratumumab were not yet available. This may impact the interpretation of our findings and their generalizability to the current patient population. Future studies are needed to validate the prognostic value of Gal-3 in patients treated with newer therapies, such as daratumumab, to establish its role in the context of contemporary treatment strategies and provide a more comprehensive understanding of its clinical utility. Secondly, our study did not monitor Gal-3 levels longitudinally, which is another limitation. Serial measurements of Gal-3 levels during follow-up could provide valuable insights into the dynamic changes of this biomarker and its potential impact on prognosis. Future prospective studies should consider incorporating longitudinal monitoring of Gal-3 levels to further assess its prognostic value and explore its potential role in guiding treatment decisions. Lastly, we used an interventricular septal thickness > 12 mm as the diagnostic criterion for defining cardiac involvement in both male and female patients. Given the gender differences in cardiac structure and size, a lower diagnostic threshold may be more appropriate for women. However, there is currently no consensus on gender-specific diagnostic criteria in previous studies and expert consensus. We chose to adhere to the established consensus criteria to ensure consistency with previous studies and maintain comparability, but we acknowledge that the lack of gender-specific diagnostic criteria is a potential limitation of our study. Future research should focus on validating and refining gender-specific diagnostic criteria for cardiac involvement in AL amyloidosis to improve diagnostic accuracy and prognostic stratification.

Furthermore, the observational design of this study prevents the direct inference of causal relationships from the noted associations. The measurements of Gal-3 were conducted on stored samples, which may have affected the accuracy of the results. The optimal cut-off for Gal-3 threshold identified in the cardiac AL amyloidosis population—15.154 ng/mL—was lower than that reported in a previous study (20.24 ng/mL) [4]. The relatively modest sample size might impinge on the robustness of our findings and restrict their generalizability. The limited number of patients could potentially undermine the power of this study to discern significant associations and might circumscribe the exploration of the utility of Gal-3 in AL amyloidosis. Whilst our results contribute to insights into the potential role of Gal-3 as a prognostic marker in cardiac AL amyloidosis, these findings should be interpreted with circumspection. Future large-scale studies with randomizations will be pivotal in investigating the role of Gal-3 in risk stratification and prognosis for patients with cardiac AL amyloidosis.

## 5. Conclusions

This study demonstrates that serum Gal-3 is an independent prognostic marker for survival in patients with cardiac AL amyloidosis. A cut-off value of 15.154 ng/mL was identified, and patients with Gal-3 levels above this threshold had significantly shorter overall survival. The prognostic value of Gal-3 remained significant after adjusting for recognized risk factors. These findings suggest that Gal-3 could potentially enhance the prognostic capabilities of the current staging systems and improve the management of cardiac AL amyloidosis. However, further validation in larger prospective studies is warranted to confirm the prognostic role of Gal-3 and explore its potential utility in clinical practice.

## Figures and Tables

**Table 1:** Comparison of baseline characteristics between patients with low and high galectin-3 levels.

Variable	All Patients N = 60	Low Gal-3 N = 37	High Gal-3 N = 23	*p*-Value
Age (years)	60.02 ± 9.72	58.62 ± 9.36	62.26 ± 10.07	0.160 ^a^
Gender (male, %)	60.00	54.05	69.57	0.233 ^b^
NYHA class (I/II, %)	60.00	48.65	34.78	0.292 ^b^
Mayo 2012 stage (I/II, %)	31.67	29.73	34.78	0.683 ^b^
SBP (mmHg)	108.8 ± 17.16	109.81 ± 18.09	107.17 ± 15.80	0.567 ^a^
DBP (mmHg)	68.33 ± 11.54	70.76 ± 12.35	64.43 ± 9.05	0.038 ^a^
Laboratory tests				
Creatinine (mg/dL)	80.00 (68.75, 106)	77.00 (67.00, 91.00)	89.00 (76.50, 169.50)	0.044 ^c^
eGFR (mL/min)	83.22 ± 18.87	86.39 ± 16.62	78.12 ± 21.42	0.099 ^a^
ALT (U/L)	17.00 (12.50, 24.50)	17.50 (14.75, 25.00)	15.00 (11.50, 22.00)	0.129 ^c^
ALP (U/L)	83.50 (64.00, 122.25)	83.00 (64.00, 122.00)	91.00 (67.50, 124.00)	0.568 ^c^
24-h urine protein (g)	0.57 (0.14, 2.84)	0.33 (0.12, 1.84)	2.13 (0.34, 5.86)	0.028 ^c^
dFLC (mg/dl)	271.90 (122.38, 471.55)	275.25 (124.63, 442.51)	226.60 (101.28, 775.00)	0.975 ^c^
cTnI (μg/L)	0.08 (0.03, 0.14)	0.07 (0.03, 0.11)	0.10 (0.04, 0.21)	0.181 ^c^
NT-proBNP (pg/mL)	3589.50 (2076.50, 6091.75)	4002.00 (2657.00, 5282.00)	2937.00 (1748.50, 13,239.00)	0.970 ^c^
Electrocardiogram				
Low voltage (%)	33.33	29.73	39.13	0.575 ^b^
AVB (%)	13.34	16.22	8.70	0.698 ^b^
LBBB (%)	16.67	10.81	26.09	0.161 ^b^
Atrial fibrillation (%)	26.67	18.92	39.13	0.133 ^b^
Echocardiography				
IVS (mm)	13.00 (11.78, 16.00)	14.00 (12.00, 17.00)	13.00 (11.00, 14.75)	0.076 ^c^
LVEF (%)	57.25 ± 10.50	56.73 ± 10.73	58.13 ± 10.30	0.633 ^a^
LAAD (mm)	40.05 ± 5.29	40.78 ± 5.12	39.40 ± 5.62	0.543 ^a^
Pericardial effusion (%)	86.67	91.89	78.26	0.240 ^b^
Comorbidities				
Heart failure (%)	65.00	70.27	56.52	0.404 ^b^
Hypertension (%)	30.00	24.32	39.13	0.257 ^b^
CAD (%)	6.67	2.70	13.04	0.153 ^b^
Diabetes mellitus (%)	11.67	13.51	8.70	0.697 ^b^
PAD (%)	3.33	5.41	0	0.519 ^b^
Carpal tunnel syndrome (%)	1.67	0	4.35	0.383 ^b^
Treatment response (no, %)	53.33	54.05	52.17	0.887 ^b^
Mortality (death, %)	38.33	29.73	52.17	0.082 ^b^
Gal-3 (ng/mL)	14.28 ± 5.29	10.91 ± 2.93	19.70 ± 3.32	<0.001 ^a^

Data are presented as number (percentage), mean ± SD, or median (interquartile range), as appropriate; ^a^ The results of Student’s *t*-tests; ^b^ the results of chi-square tests; ^c^ the results of Mann–Whitney U tests. Reference values: creatinine, 59–104 mg/dL (male), 45–84 mg/dL (female); ALT, 7–40 U/L; ALP, 35–135 U/L; 24-h urine protein, 0–0.2 g; Kappa FLC, 3.3–19.6 mg/dL; Lambda FLC, 5.7–26.3 mg/dL; cTnI, 0–0.056 μg/L; NT-proBNP, 0–125 pg/mL. Abbreviations: ALP, alkaline phosphatase; ALT, alanine aminotransferase; AVB, atrioventricular block; cTnI, cardiac troponin I; DBP, diastolic blood pressure; dFLC, difference between the involved and uninvolved free light chain; eGFR, estimated glomerular filtration rate; Gal-3, galectin-3; IVS, interventricular septum; LAAD, left atrial anteroposterior diameter; LBBB, left bundle branch block; LVEF, left ventricular ejection fraction; NT-proBNP, N-terminal pro-brain natriuretic peptide; NYHA, New York Heart Association; PAD, peripheral arterial disease; SBP, systolic blood pressure.

**Table 2:** Multivariate Cox proportional hazards predicting overall survival.

Variable	HR	95% CI	*p*-Value
Age > 60 years	1.598	0.629–4.061	0.325
Male Gender	1.093	0.398–3.003	0.863
NYHA class III/IV	2.749	0.899–8.410	0.076
Mayo 2012 stage III/IV	4.184	1.308–13.390	0.016
Gal-3 > 15.154 ng/mL	2.734	1.036–7.216	0.042
DBP < 60 mmHg	0.727	0.241–2.198	0.573
Creatinine: male > 104 mg/dL, female > 84 mg/dL	1.406	0.440–4.491	0.565
24-h urine protein > 0.2 g	0.886	0.320–2.449	0.815
No treatment response	4.434	1.573–12.500	0.005

DBP, diastolic blood pressure; Gal-3, galectin-3; NYHA, New York Heart Association.

## Data Availability

Data are available upon request.
